# Seasonal variation in the diel activity of a dung beetle assemblage

**DOI:** 10.7717/peerj.11786

**Published:** 2021-07-12

**Authors:** Jorge M. Lobo, Eva Cuesta

**Affiliations:** 1 Department of Biogeography and Global Change, Museo Nacional de Ciencias Naturales (C.S.I.C.), Madrid, Spain; 2 Escuela Internacional de Doctorado, Universidad Rey Juan Carlos, Móstoles, Madrid, Spain

**Keywords:** Phenology, Scarabaeoidea, Iberian Central System, Climatic preferences, Temporal rhythms

## Abstract

The seasonal and diel variations of dung beetle species were studied in an Iberian mid-mountain locality to examine the interaction between these two temporal rhythms. We assume that a seasonal variation in the diel activity would support the notion that both rhythms may assist in achieving a quick and flexible response when the climatic conditions change. Data coming from 4,104 pitfall traps placed during 15 sampling periods and totalling 30 daily sampling cycles were analysed using circular statistics and General Linear Models. A wide variety of seasonal patterns are observed, highlighting those species with a clear unimodal or spring-autumn bimodal seasonal pattern. However, a midday diel pattern is the norm in most of the species, except in the case of those exhibiting a high body weight that prefer dusk or night periods. We hypothesize that most of the dung beetle species fly at noon to promote the passive heating of their muscle activity and minimize the metabolic energy expenditure. Results only partially support the seasonal variation in diel activity. Diel preferences are mainly manifested at the time of the year in which the abundance is greater. Approximately two-thirds of the considered species exhibit a similar diel activity along their seasonal active period. As consequence, a significant portion of the dung beetle species currently inhabiting Mediterranean mid-mountains are not able to use the daily variation in climatic conditions to limit the inconveniences of climate change.

## Introduction

Periodicity in the life history of organisms is ultimately a response to the astronomical cycles, such as solar time, which regulate life cycles. Pairing the main life history events of organisms (growth, development, locomotor activities, mating, foraging, or dormancy) with the annual changes (photoperiodism) is important to anticipate and synchronise life history phases seasonally. Similarly, daily rhythms (circadian clock) are important to integrate behavioural, endocrine and physiological events in accordance with the daily changes of light, temperature or other environmental factors. Both phenological (Diamond et al., 2011; Duchenne et al., 2020) and diel cycles (Hut et al., 2012; Levy et al., 2019) may shift in response to climatic changes. Furthermore, both rhythms can be functionally related in terrestrial organisms (Reebs, 2002; Kronfeld-Schor & Dayan, 2008; Pita & Mira, 2011; Caravaggi et al., 2018), and specifically in poikilothermic animals such as insects (Bradshaw & Holzapfel, 2010; Koštál, 2011). The two rhythms could act in close cooperation in the case of insects given that photoperiodism is a function of the circadian system in many but not all situations (Saunders, 2020). Yearly and daily fluctuations in temperature, precipitation, insolation or light may interact with each other, thus affecting the amount of time with suitable climatic conditions for organisms to be active (Bradshaw & Holzapfel, 2010). However, energetic demands, foraging success, habitat use or predator risk can also affect the interaction between diel and seasonal rhythms (Reebs, 2002; Kronfeld-Schor & Dayan, 2008). For example, under seasonal cold conditions, it can be beneficial for an insect to be active during the warmest day conditions; in contrast, under the hottest annual periods, it would be convenient to avoid activities during midday (Dubruille, 2008). The variability in the environmental conditions existing across yearly and daily periods would thus jointly facilitate the coexistence of assemblages composed of species with different thermal requirements (McMunn & Hernández, 2018; Kronfeld-Schor & Dayan, 2003).

The seasonal variability of dung beetle assemblages has been studied extensively in tropical (Cambefort, 1991; Gill, 1991; da Silva et al., 2018), warm (Lumaret & Kirk, 1987; Errouissi et al., 2011; Agoglitta et al., 2012; Şenyüz, Lobo & Dindar, 2019) and cold temperate environments (Landin, 1961; Holter, 1982; Hanski, 1980; Wassmer, 1994). In tropical and subtropical regions, seasonality is basically linked with the appearance of wetter conditions (Davis, 2002; Touroult et al., 2017) but virtually disappears when the rainfall remains relatively constant over the year (Gill, 1991). In cold temperate regions, the dung beetle activity reaches a peak at early summer (Hanski & Cambefort, 1991). However, in southern Euro-Mediterranean localities, the need to cope with the summer drought generates two main peaks of activity: one in spring and another minor in autumn (Lumaret & Kirk, 1987; Cuesta, Mingarro & Lobo, 2021). Although lifespans of up to 5 years have been recorded, the adults of most small and medium-sized species have a longevity of a few months (Cultid-Medina & Martínez-Quintero, 2019), and one generation in the same year or two in some cases (Martín-Piera & López-Colón, 2000).

Diel dung beetle activities have been less studied. In northernmost European localities, the flight activity of dung beetles (basically endophagic Aphodiinae species which feed and reproduce inside dung pats) is principally diurnal (Koskela, 1979). In Mediterranean climates, the number of crepuscular-nocturnal species is higher but nevertheless lower than the number of diurnal dung beetles (Mena, Galante & Lumbreras, 1989). In tropical and subtropical environments, the number of nocturnal and diurnal species is relatively similar (Cambefort, 1991; Gill, 1991).

Species differences in diel activities are often explained by using resource partitioning arguments and the facilitation of coexistence by the probable decrease of competitive interactions (Hanski & Cambefort, 1991; Caveney, Scholtz & McIntyre, 1995; Palmer, 1995; Sowig, 1997; Krell-Westerwalbesloh, Krell & Linsenmair, 2004). Although these niche differences cannot be causally linked to the current role played by interspecific interactions (Holter, 1982; Niino et al., 2014), they can be attributed to the ghost of competition past (Connell, 1980). Seasonal and diel temporal dimensions would be able to interact with each other in dung beetles. Spatial variation in the phenology of dung beetles has been widely observed (Hanski, 1980; Hanski & Cambefort, 1991; Palmer, 1995; Errouissi et al., 2011; Cuesta, Mingarro & Lobo, 2021) as well as inter-annual phenological changes associated with climatic changes (Menéndez & Gutiérrez, 2004; Cuesta & Lobo, 2019). In addition, diel activity can be affected by temperature (Giménez-Gómez et al., 2018), the quantity of trophic resources (Martín-Piera, Sanmartín & Lobo, 1994), and mainly by the seasonality. The interaction between seasonal and diel variations has been noted but not specifically studied for the dung beetles of South Africa (Davis, 1996), subtropical and tropical America (Fincher et al., 1986; Gill, 1991; Kohlmann, 1991), southern Europe (Mena, Galante & Lumbreras, 1989), and northern Europe (Landin, 1961; Landin, 1968; Koskela, 1979) in which some species with a crepuscular or nocturnal activity during summer change to a diurnal activity during spring and autumn. Using an extensive annual survey performed in a mid-mountain Iberian locality, this study aims to describe the seasonal and diel variation of dung beetles and mainly seeks to examine the interaction between these two temporal rhythms. A seasonal variation in the diel activity of dung beetle species would support the functional link between these two rhythms as well as its interrelated capacity to achieve a quick and flexible response when the climatic conditions change. In contrast, diel activity that is minimally flexible or not flexible across the year may suggest that these two rhythms are basically independent, thus limiting the adaptive capacity of the species under climatic change scenarios.

## Methods

### Study site

The study area was a 1-ha border of a deciduous forest (*Quercus pyrenaica*, *Acer* spp. up to 15 m in height) that also included a meadow with scattered small trees and bushes (*Prunus* spp., *Crataegus monogyna*, *Rosa* spp., *Rubus* spp. up to 4 m) located near “El Ventorrillo” Biological Station (1,415–1,443 m a.s.l.) in the Sierra de Guadarrama (Madrid, Spain, Lat: 40.75°, Long: −4.02°). Vegetation belongs to the supra-Mediterranean type (Rivas-Martínez, 1982). The climate is continental cold Mediterranean with warm summers and cold winters, and rainfall is mainly concentrated from November to May. Mean daily average, minimum and maximum temperatures during the complete study period were 10.5 °C, 6.8 °C and 15.8 °C, respectively (temperatures recorded in the shade in the north face of a thick trunk using a HOBO^®^ Pendant Temperature/Light Data Logger). Precipitation during the year of study was 1,952 mm (May 2017 to April 2018 according to the data from the nearest Navacerrada meteorological station (4.27 km away at 1,888 m a.s.l.), while the average precipitation during the period 1966–2016 was 1,326 mm ±   330 (±sd).

### Field work

Sampling was performed from May 2017 to May 2018 using 23 pitfall traps at a time (CSR model; see Lobo, Martin-Piera & Veiga, 1988) baited with cattle dung free of antiparasitic treatments and separated approximately 8 m from each other. Baited pitfall traps consisted of plastic containers (15 cm depth ×  20 cm diameter) buried up to their rims in the soil and containing water with a drop of soap (to break surface tension) at the bottom to prevent individuals from escaping. To preclude beetles from going into the excrement, dung was introduced into nylon stockings that allowed the dung smell to escape. The amount of dung per nylon stocking and trap was fixed to ca. 250 g (mean = 255.1 g, sd = 13.2, *N* = 713; 99.9% confidence interval = 253.6–256.7 g). Nylon stockings and dung were replaced every 24–36 h. Depending on air temperature, the stockings were wetted every 6–12 h by placing them in a bucket with 15 litres of 59:1 water:dung mixture for 30 s. The pitfall traps were placed in three different micro-habitats to reflect local habitat diversity: open meadows receiving full insolation in daytime (11 traps), edge of vegetation patches (hedgerows, 10 traps) and woodland edge completely covered by tree crown (two traps). Traps located in the woodland edge are near the grassland area (≈ 10 m).

**Figure 1:**
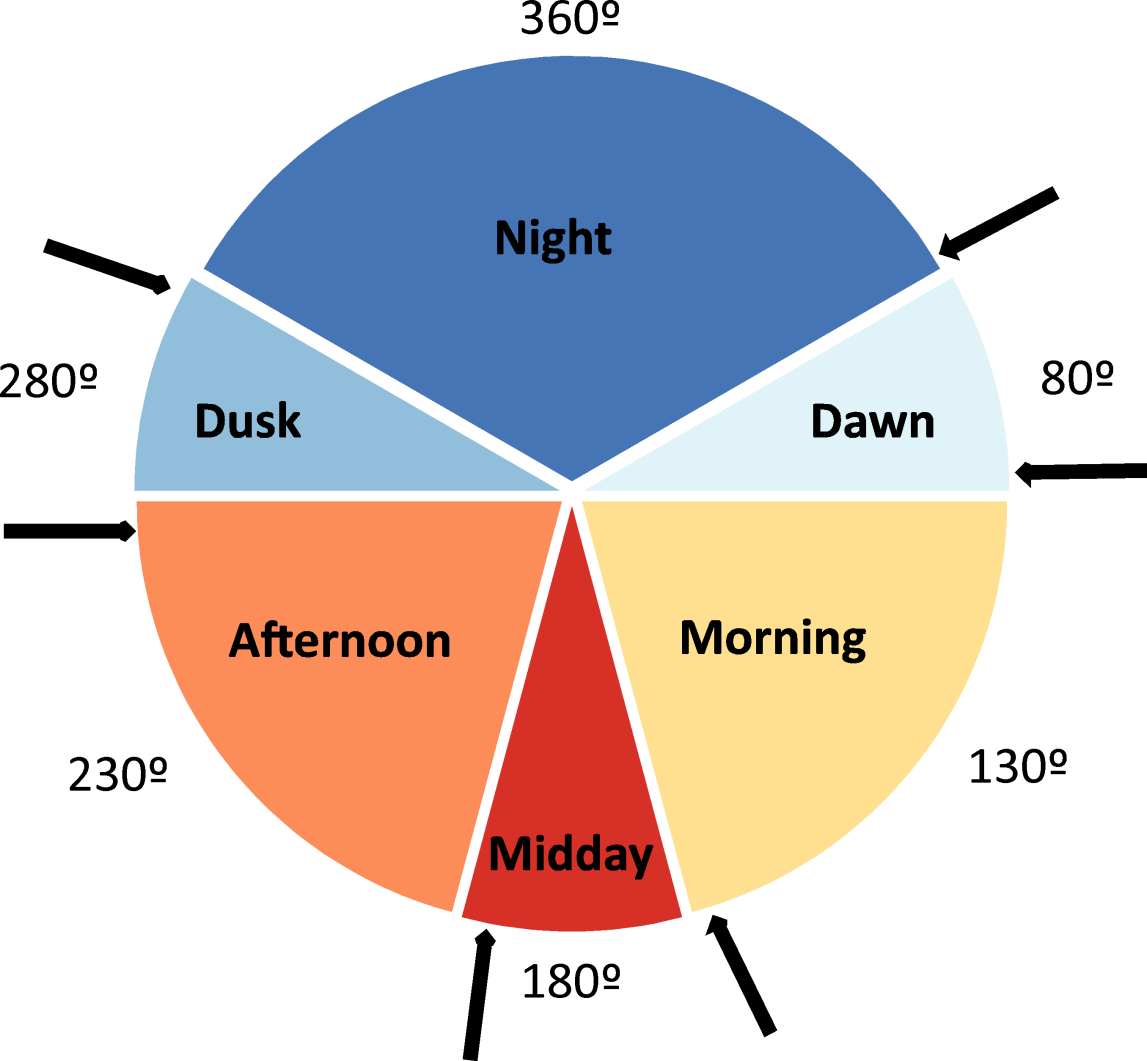
Diagram depicting the six considered daily periods. Each period has its identifying color and an angular value (360° , 80°, 130°, 180°, 230° and 280°). The arrows represent the moments at which the contents of baited pitfall traps were collected. Degrees figures are those that represent the different daily periods.

Pitfall traps were placed during 15 different sampling periods (henceforth seasonal periods; see Table 1). During each of these periods, a variable number of daily sampling cycles were performed, totalling 30 daily sampling cycles (from one to four in each sampling period; see Table 1). In each one of these daily sampling cycles, the content of each pitfall trap was collected and determined taxonomically during six occasions (Fig. 1) to determine the time of the day in which beetles actively colonize dung pats (diel activity preference). These times of capture defined six daily periods: night (from one hour after the dusk to one hour before the dawn), dawn (from one hour before dawn to one hour after dawn), morning (from one hour after dawn to two hours before midday), midday (from two hours before midday to two hours after midday), afternoon (from two hours after midday to one hour before dusk), and dusk (from one hour before dusk to one hour after dusk). In each one of these six daily periods, average air temperature was estimated in the shade using 5-min HOBO data during these periods. Sampling was not possible from December to March given the very low temperatures (local average daily temperature = 2.4 °C, average minimum temperature = −0.4 °C, average maximum temperature = 7.4 °C), the snow cover and the high amount of precipitation (61.5% of the snow and rainfall of the 12 study months in 2017–2018). Dung beetles remain inactive during this climatically unfavourable period and the few existing dung pats remain uncolonized (personal observations). We avoided windy, rainy and extreme overcast days. In two sampling periods (May 2 and May 3), only 21 pitfall traps were placed during three sampling cycles (2 less traps ×3 sampling cycles ×6 daily periods = 36). Thus, a total of 4,104 pitfall traps were considered as sampling units (6 daily periods ×30 daily sampling cycles ×23 pitfall traps = 4140 - 36 pitfall traps). The number of days that have passed since the winter solstice (21 of December) was assigned to each of these sampling units according to the corresponding sampling period.

**Table 1:** Main characteristics of the fifteen considered sampling periods. The table includes the dates in which they were made, the numbers of daily cycles (DC) performed, and the number of baited pitfall traps (BPT) placed. The number of days from the winter solstice (Day) of each sampling period is also included as well the mean air temperature of these sampling days (T^a^m), the mean air temperature at midday (T^a^mM) and the mean air temperature at night (T^a^mN).

**Period**	**Dates**	**DC**	**BPT**	**Day**	**T^a^m**	**T^a^mM**	**T^a^mN**
April1	5−6∕04∕2018	1	138	106	11.5	18.8	7.1
April2	17−19∕04∕2018	2	276	119	13.8	21.2	9.0
May1	3−5∕05∕2018	2	276	135	9.1	14.0	4.9
May2	8−9∕05∕2017	1	126	139	15.5	19.7	13.1
May3	22−26∕05∕2017	4	528	156	18.2	23.1	15.7
June1	5−9∕06∕2017	4	552	170	16.7	21.0	14.2
June2	21−23∕06∕2017	1	138	184	21.9	27.5	19.6
July	20−22∕07∕2017	2	276	213	19.4	24.5	16.2
August	4−8∕08∕2017	4	552	230	23.2	27.8	19.7
September1	5−7∕09∕2017	2	276	260	19.4	24.5	16.4
September2	20−22∕09∕2017	2	276	275	17.1	21.2	15.0
September3	28−29∕09∕2017	1	138	282	17.4	22.4	14.8
October	19−20∕10∕2017	2	276	303	10.4	13.4	6.4
November1	10−11∕11∕2017	1	138	325	8.4	14.8	5.1
November2	29−30∕11∕2017	1	138	344	0.2	3.9	−2.8

### Species selection

All beetles were taxonomically identified in the nearby “El Ventorrillo” field station. The revision of 23 traps and beetle collection usually took between 20 to 45 min per trapping event. After identification, the beetles collected in the first half of the annual survey were marked with a very small drop of pink nail polish and released 180 m away from the centre of the sampling area to estimate if activity estimations can be partially biased by the repeated collection of the same individuals. Of the approximately 4,000 individuals marked, only one individual belonging to the species *Onthophagus vacca* was trapped again in the next collection events (≈0.025%).

In total, the complete survey allowed to collect 15.182 individuals belonging to 53 species. *Aphodius fimetarius* (Linnaeus, 1758), *A. pedellus* (De Geer, 1774) = *A. cardinalis* Reitter, 1892 (Miraldo et al., 2014; Fery & Rössner, 2015) are treated as cryptic species belonging to a species complex with unclear morphological differences (Bordat, 2002; Dellacasa & Dellacasa, 2003). *Onthophagus vacca* (Linnaeus, 1767) and *O. medius* (Kugelann, 1792) are similarly treated as cryptic species belonging to a species complex difficult to differentiate by using morphological characters (Roy et al., 2016). From now on and whenever mentioned, we shall refer to *A. fimetarius* and to *O. vacca* as these species complexes. The data of the 34 species with more than 10 collected specimens were selected. These species were grouped according to their high taxonomical rank as Scarabaeinae (14 species), Aphodiinae (18 species) and Geotrupidae (2 species), and their trophic behaviour (Tonelli, 2021). Scarabaeinae and Geotrupidae species dig vertical tunnels in the ground in which they bury the dung (hypophagic behaviour). Aphodiinae species are smaller in size and live and feed inside the excrement (endophagic behaviour). Due to the tunneller behaviour of *Colobopterus erraticus* and *Teuchestes fossor* in some occasions, these two Aphodiinae species were also considered as hypophagic (Rojewski, 1983; Zunino & Barbero, 1990)*.* Fresh biomass of each of these species was estimated using the length-to-body weight relationship for dung beetles (Lobo, 1993; *r* = 0.989, *P* < 0.001). Body length was obtained from the literature (Baraud, 1977) and original descriptions of the species when necessary.

### Statistical treatment

General Linear Models (GLMs) were used to relate the mean number of collected individuals belonging to each species with the considered seasonal (S; *n* = 15) and daily periods (D; *n* = 6) following a full factorial design (i.e., considering S, D and SxD). Abundance data were transformed by ln *x* + 1 (zero data were always included). In these analyses, the explanation of the additive main effects was obviated when two-way interactions showed a relevant effect. Type III sums of squares were used to estimate the partial effect of each explanatory factor once the effects of the other factor were controlled for. Additionally, circular statistics and graphs were also used to estimate and visualize both seasonal and diel variations in occurrence (see Jammalamadaka & SenGupta, 2001**)**. Thus, seasonal and diel periods are considered as qualitative independent levels in GLM analyses but as quantitative continuous in the case of circular statistics. Each seasonal period is identified by a number that reflects the days since the winter solstice (each day thus represents 0.9863° of the complete 360° circular range). Each daily period is also identified by an angle value (night =360°, dawn = 80°, morning = 130°, midday = 180° , afternoon = 230°, and dusk = 280° ; see Fig. 1). The mean angle (µ) and its length (*r*) were calculated as circular statistic metrics. Mean angle measures the general seasonal or daily preference (except in the case of bimodal or multimodal patterns), while length measures the intensity of concentration of the observations around the mean angle ranging from zero (uniformly dispersed observations) to one (clustered observations around the mean). Rao’s spacing test was used to examine whether data are uniformly distributed around the circle (Mardia & Jupp, 2000), and Hotelling’s test was performed to examine if there is a common mean angle in the data derived from the different daily periods (Zar, 2010). A low probability indicates that the daily frequency of a species does not differ seasonally. We assume that the results of the circular statistics tests are hardly conditioned by the scarcity of species data that could have been collected during the winter unfavourable months.

The use of the terms “statistically significant” and “statistically non-significant” have been abandoned following recent recommendations (Halsey, 2019) thus considering *P*-values as indicators of the strength of the evidence. Thus, Bonferroni corrected *P*-values for multiple comparisons (34 species; 0.05/34 = 0.0015) were considered to identify “strong evidence” of relationships, whereas relationships with *P*-values from 0.05 to 0.0015 were considered “weak evidence”. StatSoft’s STATISTICA v12.0 and Oriana v.4 (Kovach, 2011) were used for these analyses.

## Results

### Seasonal patterns

**Figure 2:**
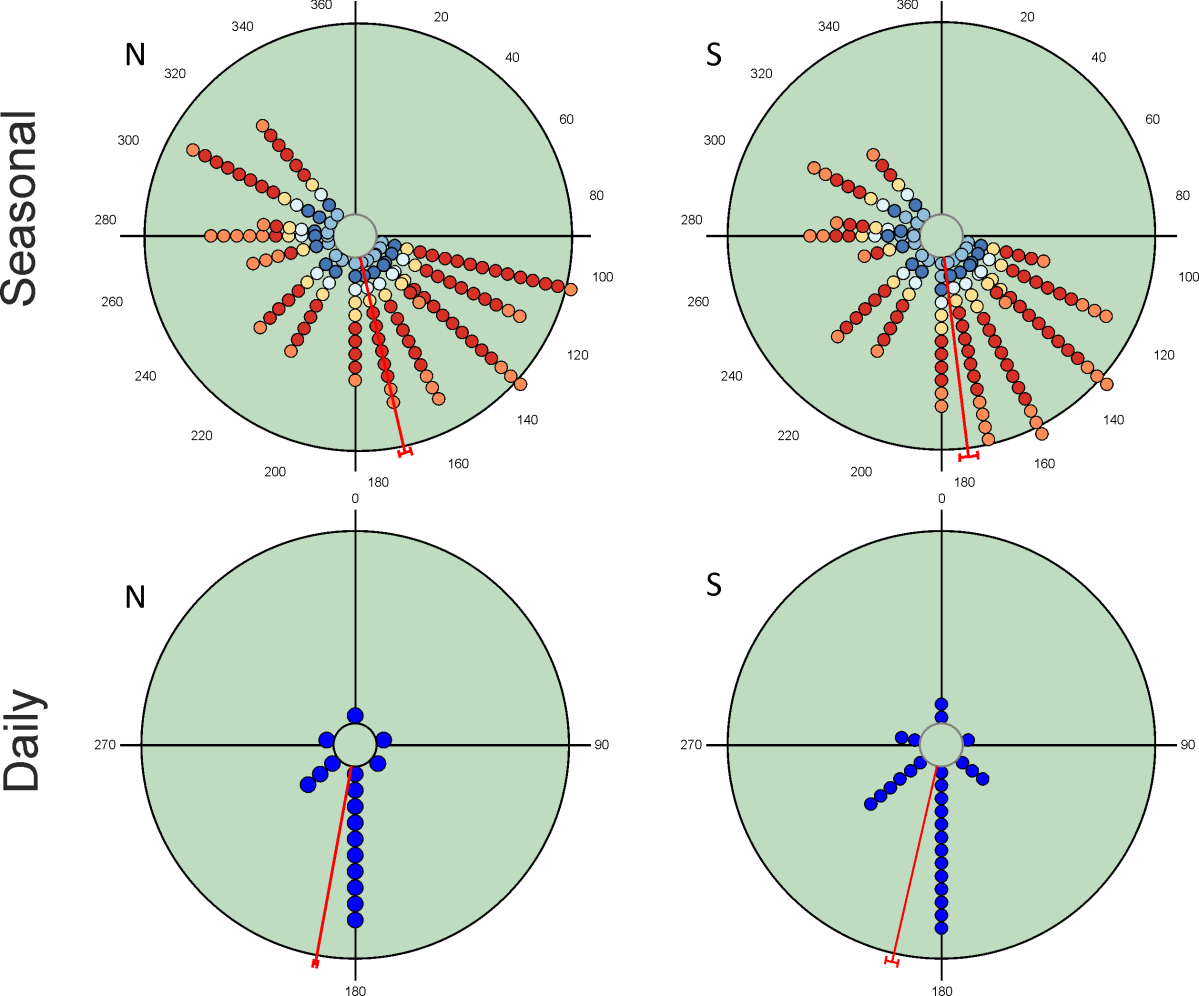
Circular diagrams representing the variation in the total number of collected individuals (N) and species richness (S) for seasonal and daily periods. In the case of seasonal variations, colours of the circles match those of Fig. 1 in representing daily periods, and the 0° corresponds to the day of the winter solstice. Daily periods follow a circular pattern such that 360° (= 0°) corresponds to the night period and 180° to midday (see Fig. 1). Each point represents the same number of individuals or species to improve the figure display. Red lines represent the value of the mean vector (±95% CI).

Seasonal variation in total abundance and species richness per pitfall trap exhibit an almost continuous pattern of occurrence from the beginning of spring to mid-autumn (Fig. 2). Mean angle values are located at the end of spring with small vector lengths values due to the temporal dispersion of the observations. However, the values of the Rao’s spacing test show that abundance and species richness data are not uniformly distributed seasonally (Table 2). This seasonal pattern is the consequence of the mixture of species with different seasonal preferences (Table 3). There are 19 species with a clear unimodal seasonal pattern; eight spring species (≈24%), four autumn species (≈12%), and seven species mainly appearing during summer (≈21%). Only three species exhibit a wide or multimodal seasonal occurrence (*O. similis*, *O. taurus* and *A. fimetarius*; Table 3 and Fig. S1), whereas the remaining 12 species (≈35%) display a well-defined spring-autumn bimodal pattern (*n* = 6), an imprecise spring-autumn bimodal pattern (*n* = 5), or a summer-autumnal bimodal pattern (*P. borealis*) (Table 3 and Fig. S1). Non-unimodal and unimodal species can be clearly differentiated by the lengths of their mean vectors (mean ±  95% CI; 0.729 ±  0.06 and 0.941 ±  0.05, respectively; Table 3).

**Table 2:** Main circular statistics of total abundance and species richness. Mean angle (µ±95% CI) and its length (*r*) of the abundance (N) and species richness (S) of all the considered observations (*n* = 4104) as well as values of the Rao’s spacing test to examine whether the data are uniformly distributed.

	Seasonal		Daily	
	N	S	N	S
µ	167.1° ± 1.5	173.0° ± 2.4	190.4° ± 0.6	193.0° ± 1.7
*r*	0.406	0.534	0.816	0.690
Rao’s Spacing test	359.7; *p* < 0.01	358.5; *p* < 0.01	359.9; *p* < 0.01	359.4; *p* < 0.01

**Table 3:** Circular and GLM statistics of each one of the species. The higher taxonomical rank (F = Family/Subfamily; S = Scarabaeinae, A = Aphodiinae, G = Geotrupidae), number of collected individuals (N) and body weight (BW; fresh biomass in mg) of dung beetle species. The mean angle (µ) and its length (*r*) of each species was included both for the seasonal and daily variations. Length values are higher in those species with a unimodal pattern. Mean angle values do not accurately reflect the mean seasonal occurrence of the species with a bimodal pattern. Seasonal patterns (SP) include the following: W/M = wide or multimodal seasonal pattern, U = seasonal unimodal pattern, B = clear seasonal bimodal pattern, b = imprecise bimodal seasonal pattern. Main types of seasonal occurrence (SO) are: Su=summer, S =spring, eS= early spring and A = autumn. Main types of daily occurrence (DO) include the following: N = night, M = midday, A= afternoon and D = dusk. General Linear Models using seasonal (S; *n* = 15) and daily (D; *n* = 6) factors were built to explain the variation in the number of collected individuals per pitfall trap for each species. R^2^ ×100 is the explanatory capacity and S ×D is the *F_(70, 4014)_* value of the interaction between the two factors which have all an associated P-value less than 0.0001 except in the case of *C. schreberi*. Hotelling’s test values (H) to examine whether the six daily periods exhibit a similar seasonal mean angle for each species.

				**Seasonal**					**Daily**					
**F**	**Species**	**N**	**BW**	***r***	µ	**SP**	**SO**		***r***	µ	**DO**	***R***^2^ × 100	**SxD**	**H**
S	*Copris lunaris* (Linnaeus, 1758)	27	143.85	0.747	204.8°	U	Su		0.795	335.6°	N	16.7	8.03	>100000^****^
S	*Euoniticellus fulvus* (Goeze, 1777)	666	15.86	0.952	217.7°	U	Su		0.966	181.6°	M	31.3	15.62	6256.28^***^
S	*Caccobius schreberi* (Linnaeus, 1758)	19	2.57	0.911	179.5°	U	S-Su		0.841	200.5°	A	2.7	0.91^ns^	408.19^**^
S	*Onthophagus coenobita* (Herbst, 1783)	111	10.73	0.787	159.1°	B	S-A		0.926	183.4°	M	14.7	5.01	89.50^*^
S	*O. fracticornis* (Preyssler, 1790)	167	13.12	0.514	143.5°	B	S-A		0.956	184.4°	M	20.9	8.06	6.98^ns^
S	*O. grossepunctatus* Reitter, 1905	89	1.59	0.978	139.5°	U	S		0.902	172.5°	M	16.0	6.62	19216.89^****^
S	*O. joannae* Goljan, 1953	54	2.26	0.936	140.9°	b	S-A		0.933	173.9°	M	12.0	4.36	12685.69^*^
S	*O. lemur* (Fabricius, 1781)	114	5.39	0.970	151.9°	U	S		0.942	179.4°	M	22.5	9.59	15333.57^****^
S	*O. opacicollis* Reitter, 1893	36	5.39	0.523	184.8°	B	S-A		0.879	177.6°	M	5.9	2.24	6.36^ns^
S	*O. ovatus* (Linnaeus, 1767)	31	2.26	0.950	233.5°	U	Su		0.928	180.4°	M	15.2	6.98	955.28^***^
S	*O. similis* (Scriba, 1790)	1926	3.10	0.658	148.3°	W/M	S-Su-A		0.933	183.3°	M	44.4	15.08	245.04^****^
S	*O. stylocerus* Graëlls, 1851	30	28.59	0.983	134.0°	U	S		0.944	175.3°	M	14.6	6.59	>100000^****^
S	*O. taurus* (Schreber, 1759)	156	14.17	0.841	203.0°	W/M	S-Su-A		0.948	186.2°	M	16.4	5.05	1369.55^*^
S	*O. vacca* (Linnaeus, 1767)	649	22.49	0.957	139.1°	U	S		0.959	180.6°	M	36.7	19.27	16628.51^****^
A	*Acrossus depressus* (Kugelann, 1792)	62	8.66	0.952	138.7°	U	S		0.951	180.7°	M	21.1	11.55	440.27^**^
A	*Agrilinus constans* (Duftschmid, 1805)	55	3.10	0.854	116.3°	b	eS-A		0.853	191.3°	M	26.4	14.31	189.82^**^
A	*Aphodius fimetarius* (Linnaeus, 1758)	171	5.39	0.642	159.9°	W/M	S-Su-A		0.886	188.6°	M	23.7	10.08	23.61^*^
A	*Aphodius foetidus* (Herbst, 1783)	233	4.13	0.891	136.9°	b	S-A		0.795	186.4°	M	22.4	7.08	1851.35^****^
A	*Bodilopsis rufus* (Moll, 1782)	99	4.13	0.839	205.4°	U	Su		0.591	328.6°	N	13.7	4.18	339.04^****^
A	*Colobopterus erraticus* (Linnaeus, 1758)	1834	4.13	0.954	170.5°	U	S-Su		0.934	181.4°	M	38.3	16.98	2365.29^****^
A	*Esymus pusillus* (Herbst, 1789)	50	1.08	0.995	133.7°	U	S		0.824	188.2°	M	18.5	7.34	>100000^****^
A	*Melinopterus prodromus* (Brahm, 1790)	72	3.49	0.941	118.4°	b	eS-A		0.854	188.7°	M	25.5	13.63	1158.75^***^
A	*Melinopterus sphacelatus* (Panzer, 1798)	3152	2.57	0.828	106.5°	B	S-A		0.884	185.7°	M	66.1	54.50	292.76^****^
A	*Nimbus contaminatus* (Herbst, 1783)	990	4.13	0.983	298.7°	U	A		0.549	183.0°	M	40.7	14.30	49443.12^****^
A	*Nimbus obliteratus* (Panzer, 1823)	68	3.10	0.564	309.4°	B	eS-A		0.765	184.7°	M	11.2	4.71	3.33^ns^
A	*Nimbus proximus* Ádám, 1994	1058	2.26	0.978	307.2°	U	A		0.785	183.9°	M	48.9	31.64	14940.98^****^
A	*Otophorus haemorrhoidalis* (Linnaeus, 1758)	340	1.59	0.964	209.8°	U	Su		0.944	181.5°	M	37.3	21.69	14505.07^****^
A	*Planolinoides borealis* (Gyllenhal, 1827)	40	1.59	0.673	210.5°	b	Su-A		0.202	240.3°	A	7.3	2.64	1093.28^****^
A	*Sigorus porcus* (Fabricius, 1792)	17	3.10	0.999	270.7°	U	A		0.891	226.3°	A	6.2	2.51	–
A	*Teuchestes fossor* (Linnaeus, 1758)	31	26.44	0.915	149.2°	U	S		0.966	178.5°	M	11.4	5.52	>100000^****^
A	*Trichonotulus scrofa* (Fabricius, 1787)	65	0.69	0.982	138.5°	U	S		0.787	198.1°	A	20.5	8.92	17540.93^****^
A	*Volinus sticticus* (Panzer, 1798)	2676	1.59	0.512	210.9°	B	S-A		0.810	223.3°	A	20.1	7.47	51.61^***^
G	*Geotrupes ibericus* Baraud, 1958	32	331.00	0.884	259.5°	U	A		0.963	280.4°	D	14.7	6.45	2138.76^*^
G	*Geotrupes mutator* (Marsham, 1802)	19	205.94	0.771	284.3°	b	S-A		0.883	273.2°	D	9.2	4.04	>100000^****^

**Notes.**

* *p* ≤ 0.05, **p ≤ 0.01, ***p ≤ 0.001 and ****p ≤ 0.0001

### Diel patterns

**Figure 3:**
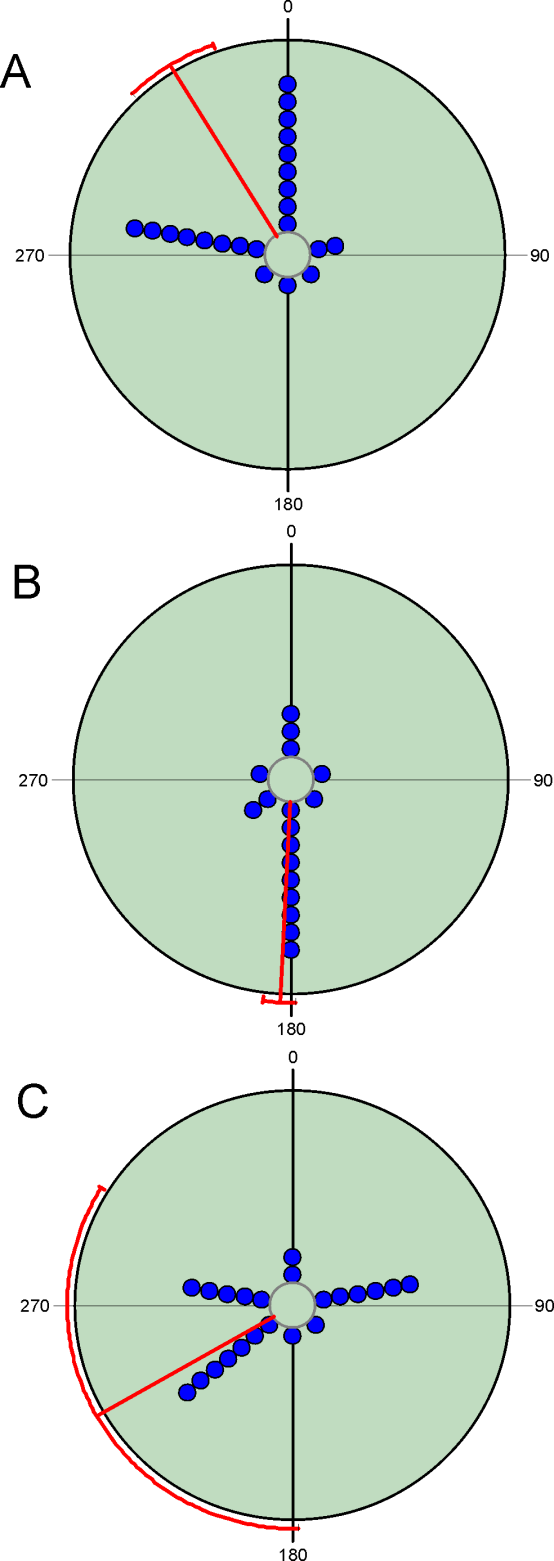
Circular diagrams representing the number of collected individuals (circles) of three selected species in the six considered daily periods. Daily periods follow a circular pattern such that 360° corresponds to the night period and 180° to midday (see Fig. 1). Each point represents the same number of individuals. Red lines represent the value of the mean vector (±95% CI). (A) = *Bodilopsis rufus*; (B) = *Nimbus contaminatus*; (C) = *Planolinoides borealis.*.

Diel variability in total abundance and species richness values is much lower than seasonal variability with mean angle figures visibly located at midday, and Rao’s spacing test values clearly indicate a non-uniform distribution of diel observations (Fig. 2; Table 2). Most of the studied species exhibit a midday diel pattern (*n* = 25; ≈73%), whereas the remaining 9 species exhibit an afternoon (*n* = 5), dusk (*n* = 2) or night (*n* = 2) pattern. Three of the four species with a preference for dusk or night periods exhibit the highest body weight (Table 3). The diel mean angles differ between the three high rank groups of species due to the dusk occurrence of the two large bodied Geotrupidae species (*F*_2,31_ = 4.27, *P* = 0.02). Vector length values are generally high except in the case of three Aphodiinae species (*B. rufus*, *N. contaminatus* and *P. borealis*; see Table 3) that are able to appear in different diel periods (Fig. 3). Thus, vector lengths also differ between the three high rank groups (*F*_2,31_ = 3.37; *P* = 0.05) due to the more dispersed character of the Aphodiinae diel observations (Table 3). The mean vector length in Aphodiinae is 0.793 ±  0.068, whereas these values are 0.918 ±  0.077 in Scarabaeinae and 0.923 ±  0.204 in Geotrupidae.

### Seasonal variation in diel occurrence

**Figure 4:**
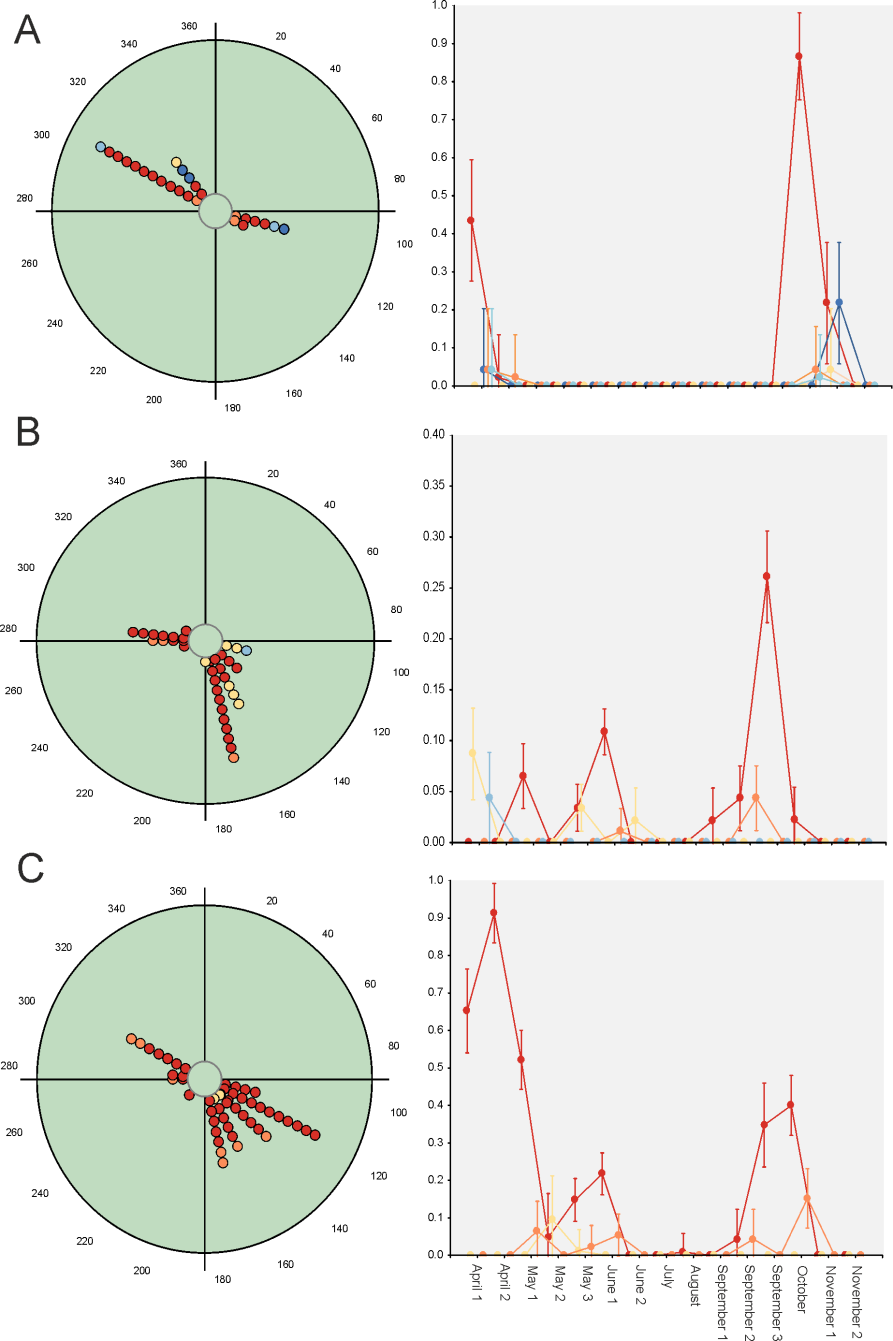
Circular diagrams representing the seasonal variation in the number of collected individuals (circles) of three selected species according to the six considered daily periods. The colours of the circles match those of Fig. 1, and 0° (=360°) corresponds to the day of the winter solstice. Each point represents the same number of individuals. At right, Seasonal × Daily plots representing the variation in the number of collected individuals per pitfall trap (±95% confidence intervals). The colours of the markers, lines and whiskers correspond to those indicated in Fig. 1. (A)=*Nimbus obliteratus*, (B)=*Onthophagus opacicollis*, (C)=*Onthophagus fracticornis.*

Hotelling’s tests (*H*) on total abundance (*H* = 22.57; *P* = 0.007) and species richness data (*H* = 96.49; *P* = 0.0004) indicate that the probability that diel preference vary seasonally is small (Fig. 2). The results of this test on species data (Table 3) provide strong evidence of a common mean seasonal angle for the occurrences of the different diel periods in the case of 21 species (62% of total). Only three species suggest that the diel occurrence would differ seasonally (*O. fracticornis*, *O. opacicollis* and *N. obliteratus*), showing a spring-autumn bimodal occurrence (Fig. 4). Weak evidence appears in 8 species (23%), indicating that *O. coenobita*, *O. joannae*, *O. taurus*, *A. fimetarius* and *G. ibericus* are candidates to vary their diel activity seasonally according to the results of the Hotelling’s tests (see Fig. S1).

**Figure 5:**
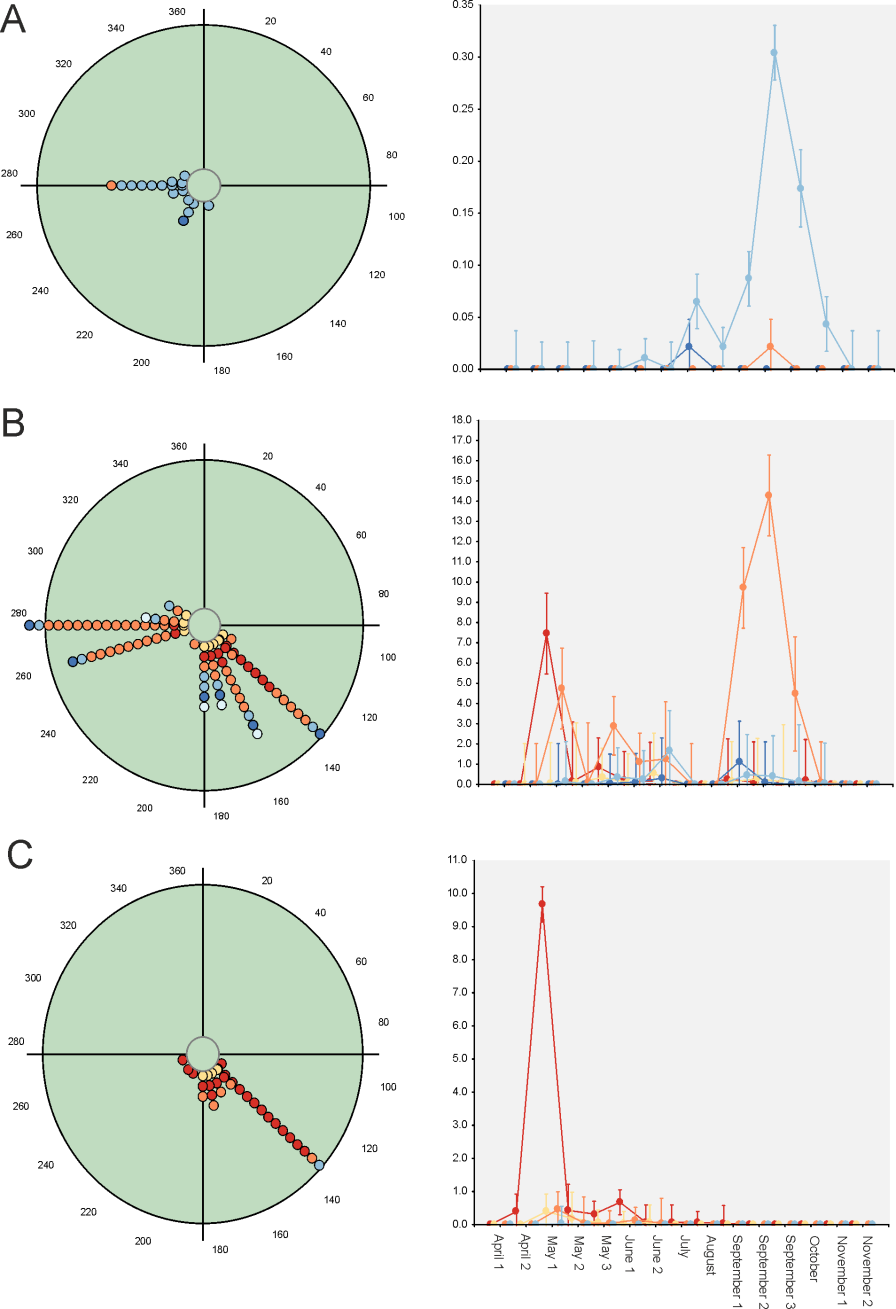
Circular diagrams representing the seasonal variation in the number of collected individuals (circles) of three selected species according to the six considered daily periods. The colours of the circles match those of Fig. 1, and 0° (=360°) corresponds to the day of the winter solstice. Each point represents the same number of individuals. At right, Seasonal ×  Daily plots representing the variation in the number of collected individuals per pitfall trap (±95% confidence intervals). The colours of the markers, lines and whiskers correspond to those indicated in Fig. 1. (A)=*Geotrupes ibericus*; (B)=*Volinus sticticus*, (C)=*Onthophagus vacca*.

Paradoxically, GLM results indicate that 33 out of 34 considered species exhibit strong evidence of a seasonal change in their diel occurrence frequency (≈97%; Table 3). Thus, according to the GLM results, dung beetle species would seasonally modify their daily occurrence because they would manifest a diel preference during the annual period in which the abundance is maximum. This can occur during the dusk or night periods (4 species: *C. lunaris, G. ibericus, G. mutator and B. rufus*), at the afternoon (4 species: *P. borealis, S. porcus, T. scrofa* and *V. sticticus*), but mainly during the midway (25 species) both in seasonal unimodal species (*E. fulvus*, *O. lemur*, O*. grossepunctatus*, *O. ovatus*, *O. taurus*, O*. vacca, C. erraticus*, *E. pusillus*, *A. depressus, O. stylocerus, N. contaminatus, N. proximus, O. haemorrhoidalis* and *T. fossor*) and in seasonal bimodal or multimodal species (*O. coenobita*, *O. fracticornis, O. opacicollis*, *O. similis*, *O. joannae, M. sphacelatus*, *M. prodromus, A. fimetarius*, *A.foetidus, N. obliteratus* and *A. constans*) (Fig. 5 and Fig. S1).

## Discussion

Dung beetles are the main species adapted to the consumption and recycling of mammal feces, an ephemeral resource. Flight is the basic activity of dung beetles in searching the resource used for its feeding and reproduction, and the results obtained in this study measure the activities of the imaginal states of these species linked with the immigration to and emigration from dung pats.

From the seasonal point of view, our results indicate that a clear bimodal pattern (spring-autumn) in the variation of assemblage abundance or species richness is not present in contrast to what commonly occurs in other Mediterranean regions (Lumaret & Kirk, 1987; Errouissi et al., 2011; Agoglitta et al., 2012; Şenyüz, Lobo & Dindar, 2019). The mountainous character of the studied locality has led to the seasonal maintenance of a diverse assemblage of dung beetles that are active during the summer but not during the winter (the mean air temperature during the winter months is 2.5 ±  0.7 °C; mean ±  95% CI). As consequence, the studied species are characterized by a wide variety of seasonal patterns both bimodal-multimodal and unimodal centred in spring, autumn or summer periods. Taking into account that the quiescence during summer drought is limited in this case and that the complete development of a dung beetle specimen would require from 30 to 80 days depending on the species (Christensen & Dobson, 1977; Rojewski, 1983; Stevenson & Dindal, 1985; Romero-Samper & Martín-Piera, 1995; Romero-Samper & Martín-Piera, 2007; Arellano et al., 2017), a significant portion of the univoltine species probably hibernate as larval and pupal stages.

Diel preferences demonstrate that greater than three-fourths of the considered species prefer midday to be active. Such midday activity can occur in typical spring species, such as *O. stylocerus*, *O. vacca* or *T. fossor*; summer species, such as *O. haemorrhoidalis* or *E. fulvus*; or even in autumnal species, such as those belonging to the *Nimbus* genus. In general, endophagic Aphodiinae species seem to exhibit a more dispersed diel rhythm than hypophagic Scarabaeinae species probably as a consequence of their greater contact with the conditions of the soil surface. Due to their high vector lengths, this pattern is even reinforced if the Aphodiinae species *Colobopterus erraticus* and *Teuchestes fossor* are considered as hypophagic. Endophagic species can quickly be active at different daily periods if the temperature and humidity conditions are optimal (Landin, 1968). The preference for the daily period at which the sun is highest in the sky suggests that the activity in many dung beetle species could be facilitated by solar radiation. Sun irradiance absorbed or transmitted dorsally propitiate the heat gain in the internal body of dung beetles (Carrascal, Jiménez-Ruiz & Lobo, 2017; Amore et al., 2017) probably as consequence of the absorbance by the exoskeleton of the ultraviolet and visible wavelengths (Alves, Hernández & Lobo, 2018). Thus, in a mid-mountain Mediterranean region, such as the one studied here, most of the dung beetle species would be flying at noon to promote the passive heating of their muscle activity and minimize the metabolic energy expenditure. This midday activity may suppose a risk of overheating if the flight or soil activities are strong but probably not if the beetle is able to thermoregulate (Gallego, Verdú & Lobo, 2018) or quickly finds a dung pat to bury in it or in the ground (see Smolka et al., 2012; Verdú, Alba-Tercedor & Jiménez-Manrique, 2012). Thus, the overheating risk would be counterbalanced by the advantage of an early arrival to the dung (e.g., to avoid competition or predation).

Interestingly, species preferring dawn or morning diel periods are not observed, a pattern which might suggest that the start of flight activity is determined by temperature after the night (Koskela, 1979; Caveney, Scholtz & McIntyre, 1995; Byrne & Dacke, 2011). The thermal influence on diel activity is also evident in the preference of large dung beetles for afternoon, dusk or night periods (see also Mena, Galante & Lumbreras, 1989), a pattern generally observed in arthropods (McMunn & Hernández, 2018; Guevara & Avilés, 2013). The nocturnal or crepuscular habits of some dung beetles have been so stable that they display specific morphological adaptions related to eyes and vision (McIntyre & Caveney, 1998; Byrne & Dacke, 2011). Flying at night may prevent overheating in larger beetles with a lower surface area to volume ratio but may require a metabolic effort to elevate body temperature for take-off. Large dung beetles can be endothermic (Caveney, Scholtz & McIntyre, 1995; Verdú, Arellano & Numa, 2006; Verdú, Alba-Tercedor & Jiménez-Manrique, 2012); however, their absence during dawn or morning periods in our study suggest that these species could take advantage of the infrared radiation accumulated in the soil after a daytime period with solar radiation. This assumption is reinforced by the fact that ventral body parts of dung beetles exhibit a comparatively increased relevance in the acquisition of heat coming from the substrate (Carrascal, Jiménez-Ruiz & Lobo, 2017), and because quick heat gains from cold conditions seem to be facilitated by the permeability of the exoskeleton to infrared radiation (Amore et al., 2017).

The interdependence between seasonal and diel rhythms has been observed in other animals (Reebs, 2002; Kronfeld-Schor & Dayan, 2008; Pita & Mira, 2011; Caravaggi et al., 2018; Bradshaw & Holzapfel, 2010; Koštál, 2011), although the widespread nature of this interaction in the different groups is overlooked. Our results partially support a seasonal variation in the diel activity of these mid-mountain Mediterranean dung beetle species. Seasonal and diel preferences may appear as interacting when both periods are considered as qualitative levels in GLM analyses. In this case, the diel predilection is mainly manifested at the time of the year in which the abundance is higher. This period should coincide with the moment at which a high probability of sexual encounter exists. Thus, those individuals far from their optimum seasonal period would be those that would not manifest marked diel preferences. However, this pattern changes when the seasonal variation is considered quantitatively and circular statistics are used. We rely more on the results of circular statistics because the temporal proximity of the sample units should be a decisive factor to consider (Jammalamadaka & SenGupta, 2001). In this case, approximately two-thirds of the considered species would have a similar diel activity along their seasonal active period. In the remaining species, a diel period is favoured during the yearly season with more active individuals (mainly midday), and comparatively more individuals can be observed at morning or evening in the periods far from the main seasonal peaks (see Fig. S1). Our results thus outline the need to analyse temporal data as those used in this study by means of circular statistics to avoid erroneous conclusions.

Hence, we may assume that the interaction between the two temporal rhythms is not a universal pattern. The seasonal variation in diel preferences seems to be the norm in the endophagic Aphodiinae species inhabiting northern Europe (Schmidt, 1935; Landin, 1961; Landin, 1968; Koskela, 1979). In this study, we also noted that endophagic Aphodiinae species exhibit a more dispersed diel rhythm. However, Mediterranean dung beetle assemblages are more complex and diverse, and their environment harbours a broader range of suitable climatic conditions for an ectotherm. The capacity of the organisms to maintain their physiological and performance traits along a complete temporal interval diminishes when the cyclic environmental fluctuations are broader (Niehaus et al., 2012). As consequence, it would be reasonable to expect increased variety of seasonal and diel responses in Mediterranean species, ranging from those able to seasonally change their diel activity to others with a clear and minimally flexible diel preference. Taking into account these results, we hypothesize that a significant portion of the dung beetle species currently inhabiting Mediterranean mid-mountains will not be able to use the daily variation in climatic conditions to limit the inconveniences of climate change.

## Conclusions

1. The studied mid-mountain dung beetle assemblage does not show the classic seasonal bimodal pattern of Mediterranean localities, and a wide diversity of seasonal patterns are observed.
2. More than three-quarters of the considered species prefer midday to be active, a pattern probably related with the use of solar radiation to propitiate the passive heating of the body.
3. The thermal influence on diel activity became evident by the fact that there were no species preferring dawn or morning diel periods, and that large dung beetles significantly prefer afternoon, dusk or night periods.
4. Endophagic species seem to exhibit a more dispersed diel rhythm than hypophagic species probably as consequence of their greater contact with the soil conditions.
5. The seasonal variation in diel activity is only partially supported by our results since around two-thirds of the considered species would have a similar diel activity throughout the year. Individuals far from their optimum seasonal period would be those that would not manifest marked diel preferences
6. Our results indicate the need to analyse temporal data by the use of circular statistics to avoid erroneous conclusions.
7. We hypothesize that most part of the mid-mountain dung beetle species inhabiting under Mediterranean conditions will not be able to use diel variations to surpass the difficulties generated by climate change.

##  Supplemental Information

10.7717/peerj.11786/supp-1 Supplemental Information 1 Circular diagrams representing the number of collected individuals (circles) of each species within the six considered daily periods Each daily period is identified by a colour (night = black, dawn = light blue, morning = yellow, midday = red, afternoon = green, and dusk = dark blue° ) and 0° corresponds to the day of the winter solstice. Each point represents the same number of individuals. At right, Seasonal x Daily plots representing the variation in the number of collected individuals per pitfall trap (±95% confidence intervals).

10.7717/peerj.11786/supp-2 Supplemental Information 2 Raw data Taxonomic composition, species richness, abundance and habitat, seasonal and daily characteristics of all the pitfall traps used in this study.

## Additional Information and Declarations

### Data Availability

The following information was supplied regarding data availability: Raw data are available in the Supplemental Files.
