# Supplementary material for: Seasonal variation in the diel activity of a dung beetle assemblage
Source: PeerJ. 2021 Jul 12;9:e11786. doi: 10.7717/peerj.11786 (PMC8280883; doi:10.7717/peerj.11786)

Copris lunaris

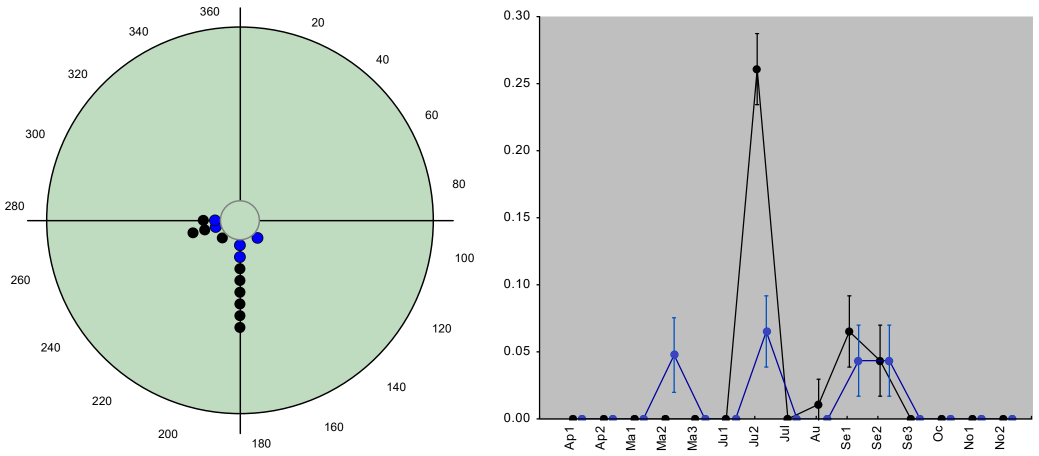

Euoniticellus fulvus

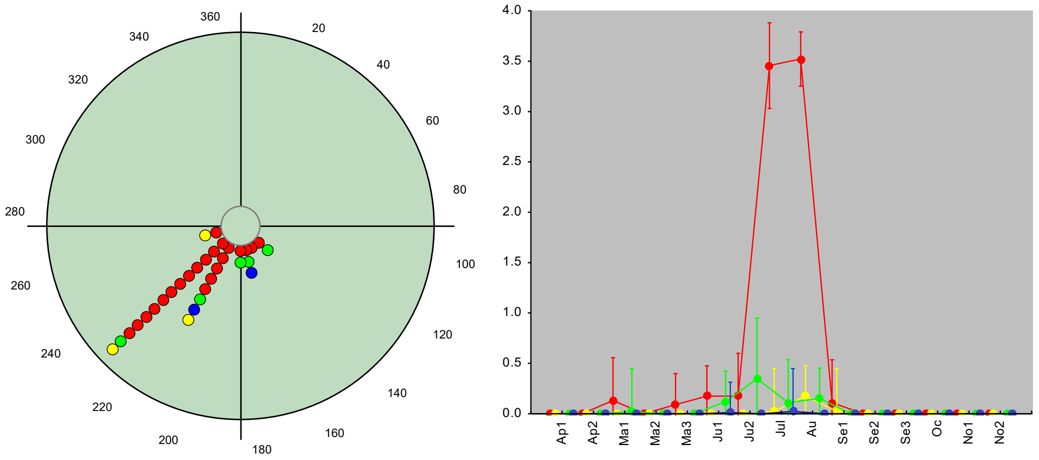

Caccobius schreberi

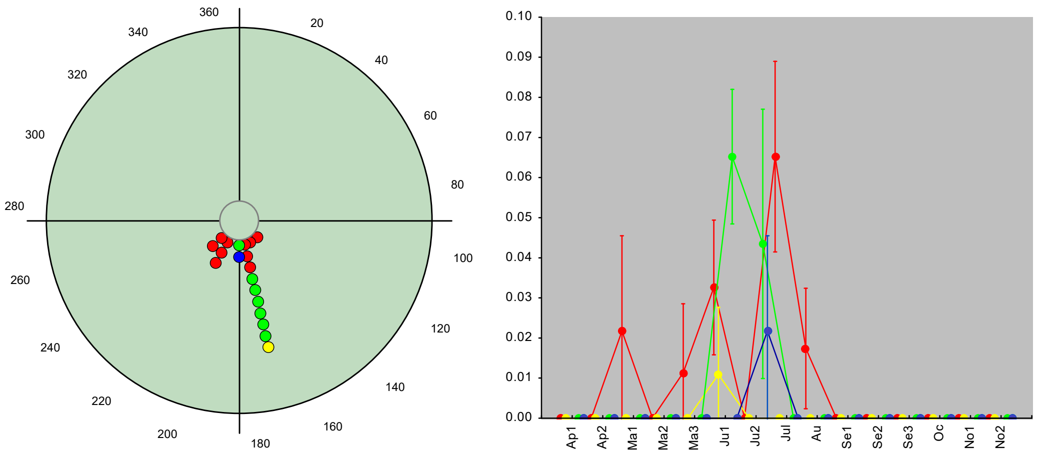

Onthophagus coenobita

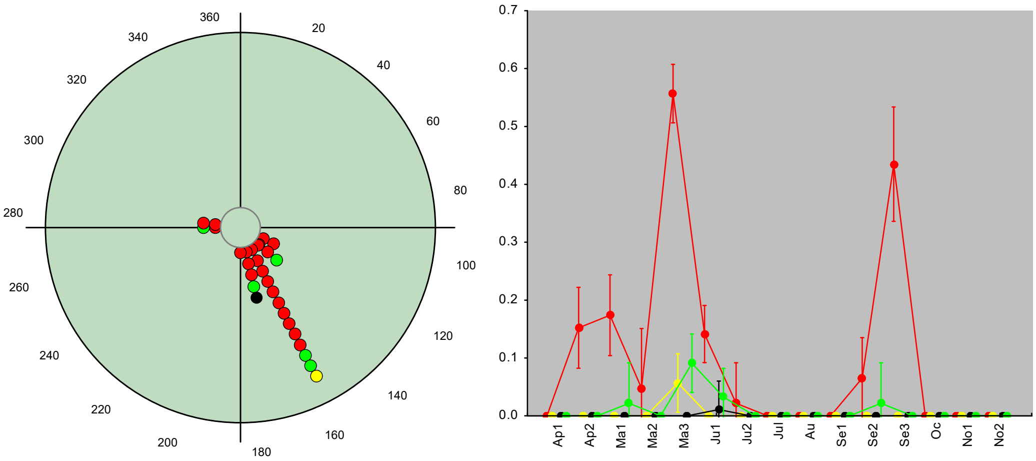

Onthophagus grossepunctatus

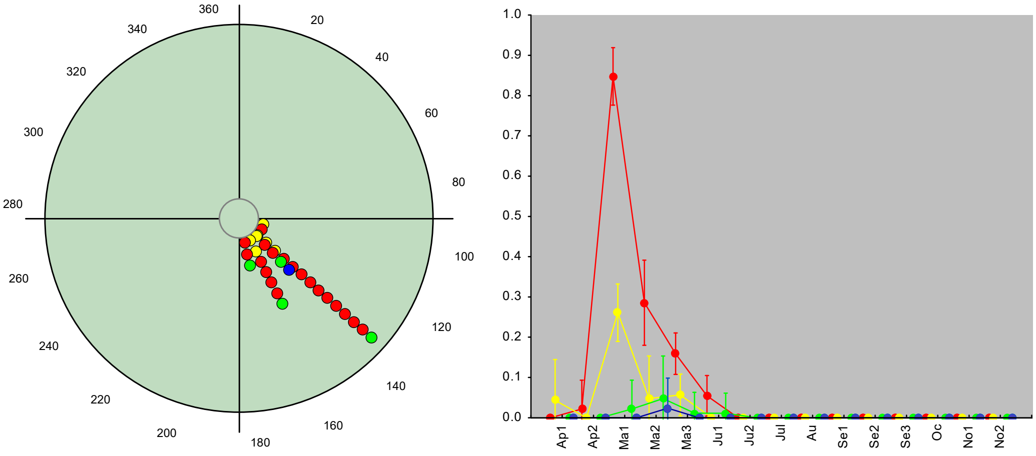

Onthophagus joannae

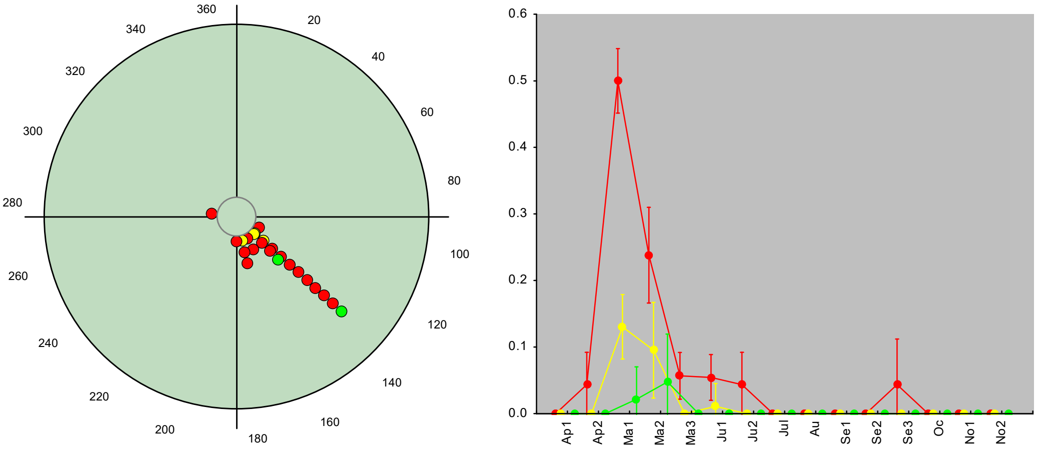

Onthophagus lemur

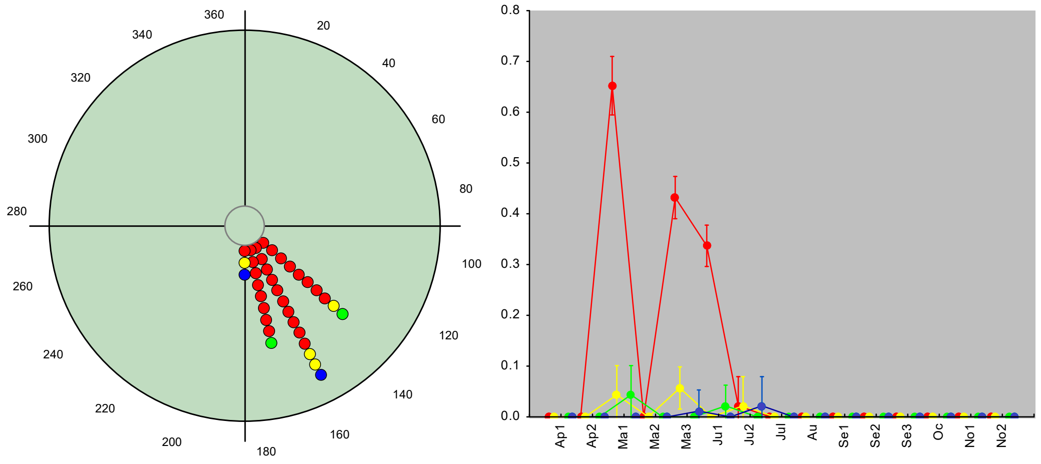

Onthophagus ovatus

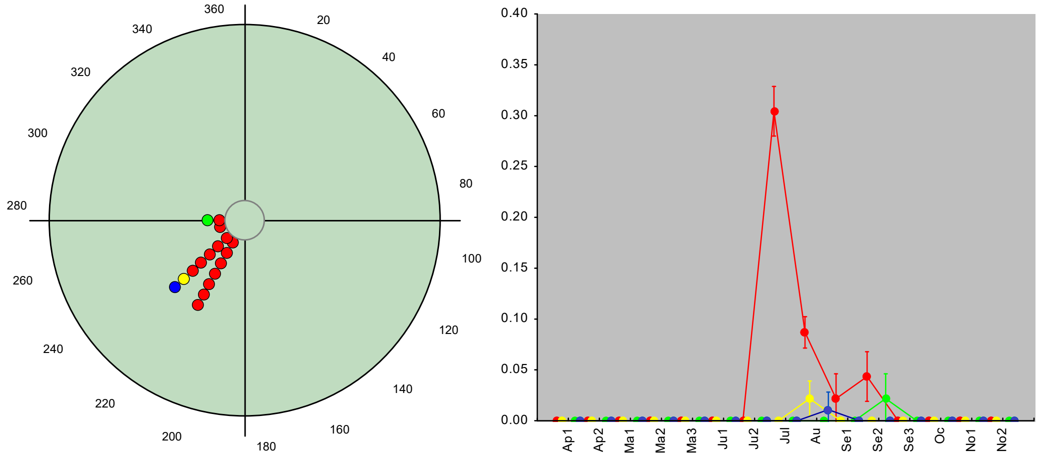

Onthophagus similis

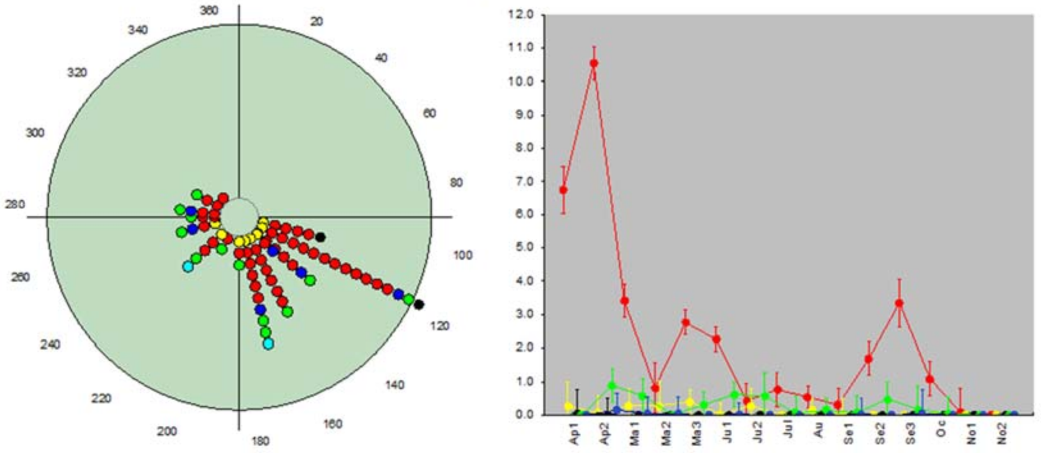

Onthophagus stylocerus

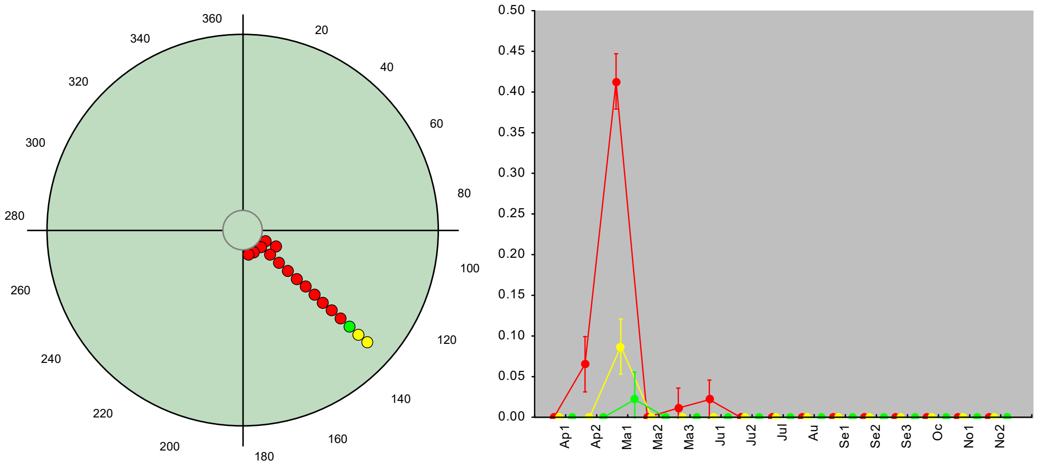

Onthophagus taurus

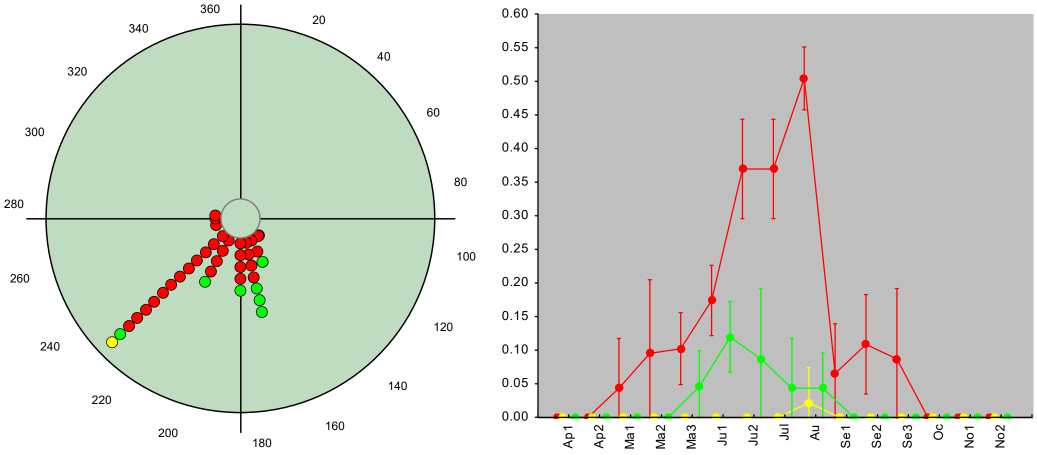

Acrossus depressus

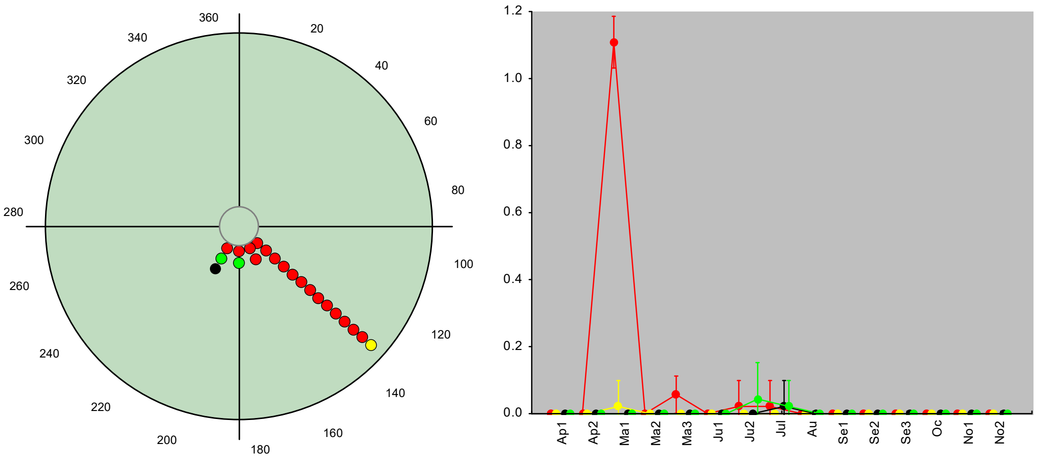

Agrilinus constans

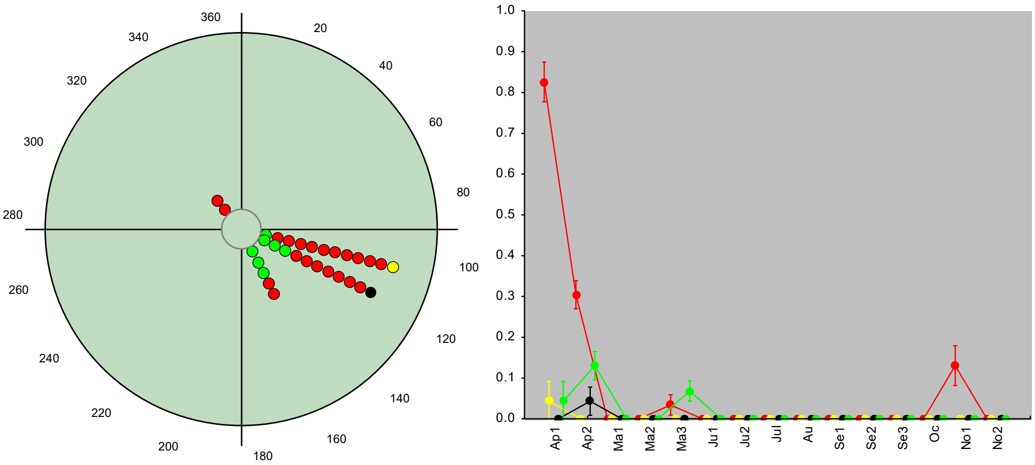

Aphodius fimetarius

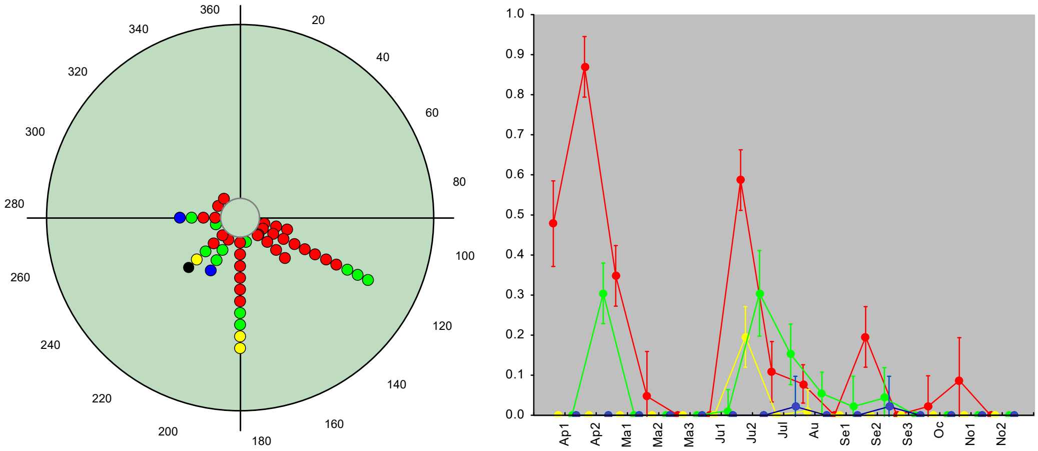

Aphodius foetidus

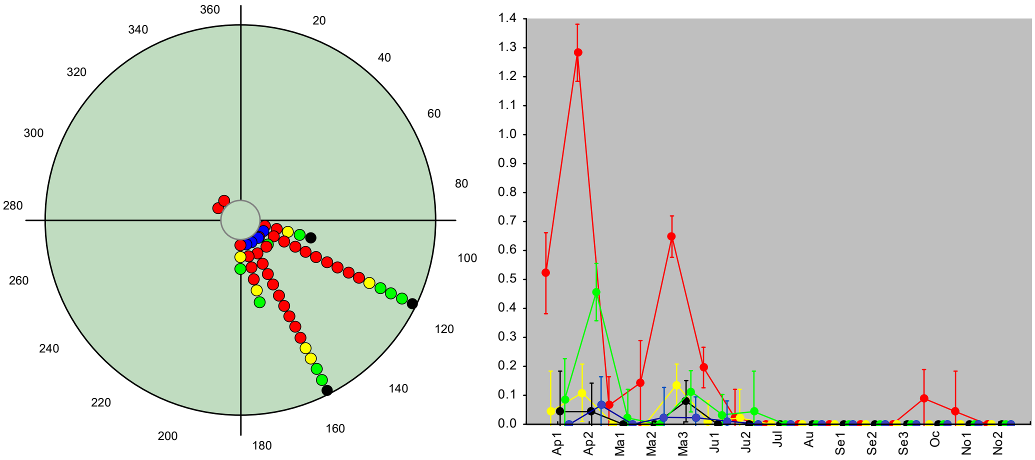

Bodilopsis rufus

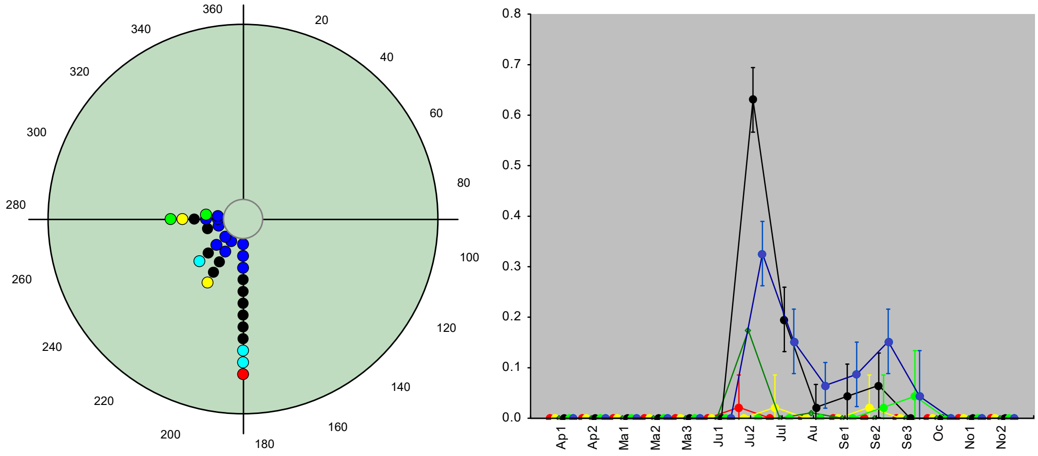

Colobopterus erraticus

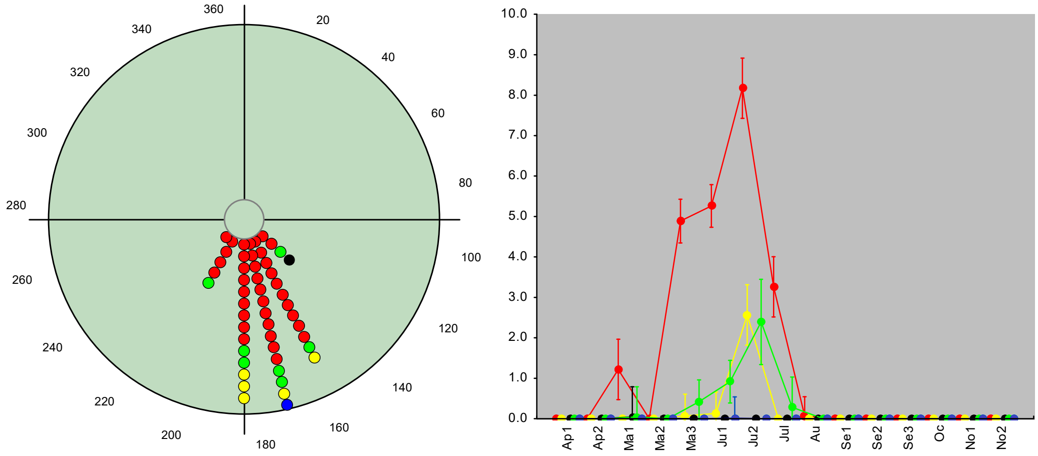

Esymus pusillus

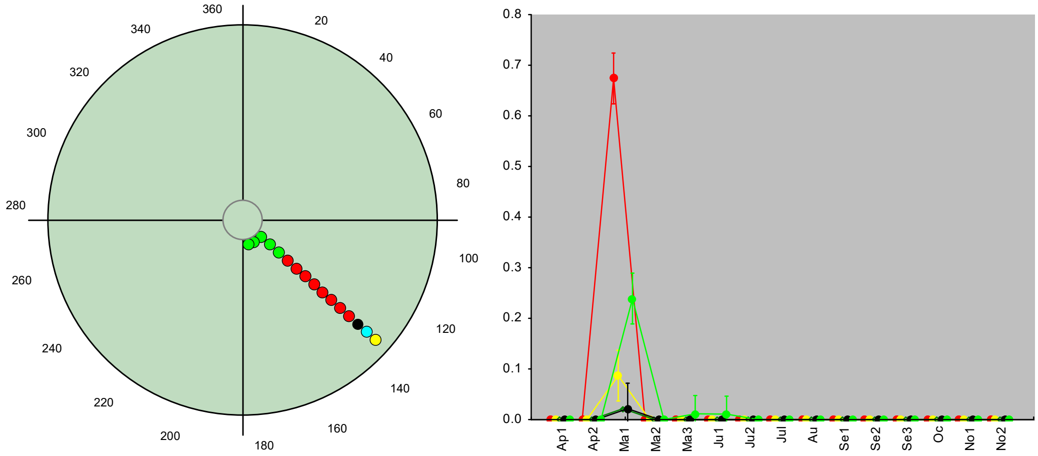

Melinopterus prodromus

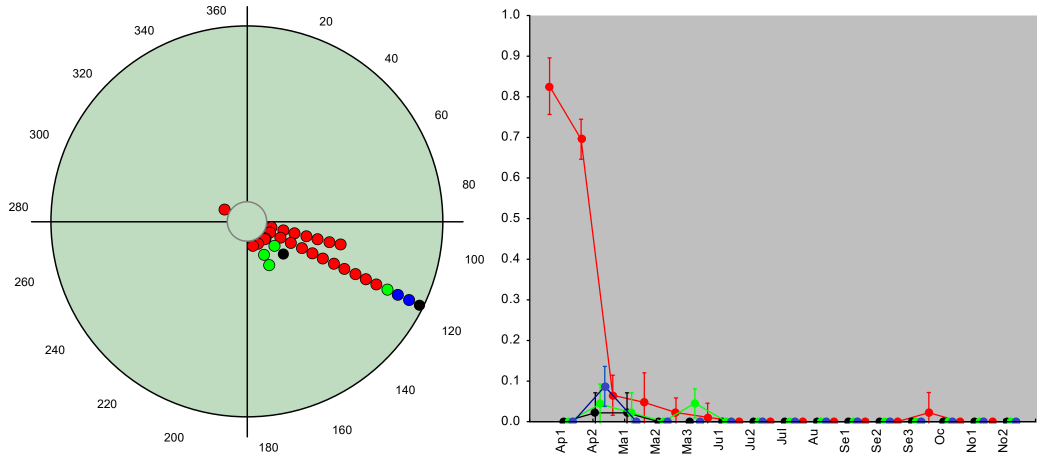

Melinopterus sphacelatus

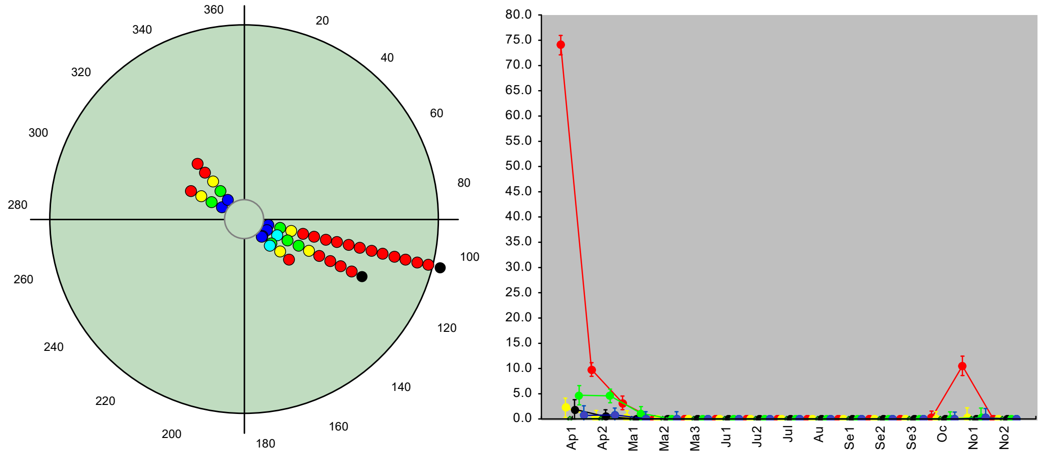

Nimbus contaminatus

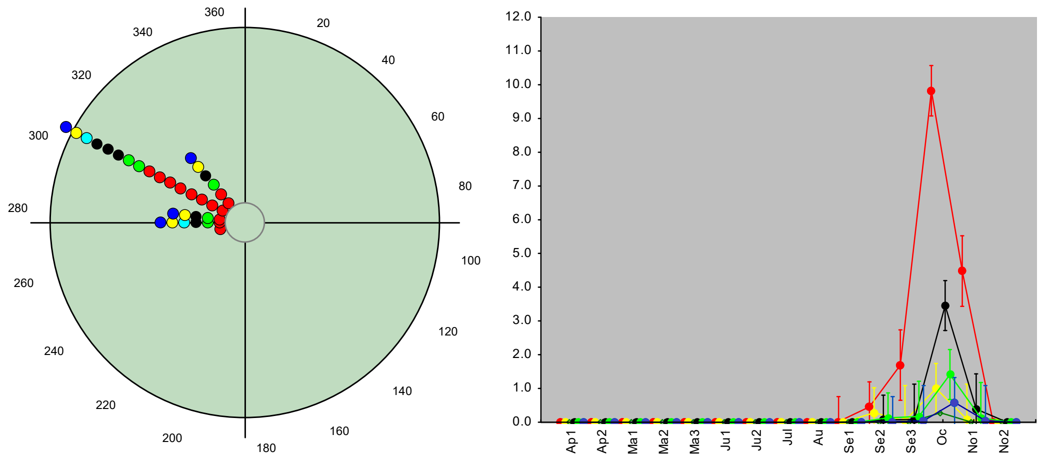

Nimbus proximus

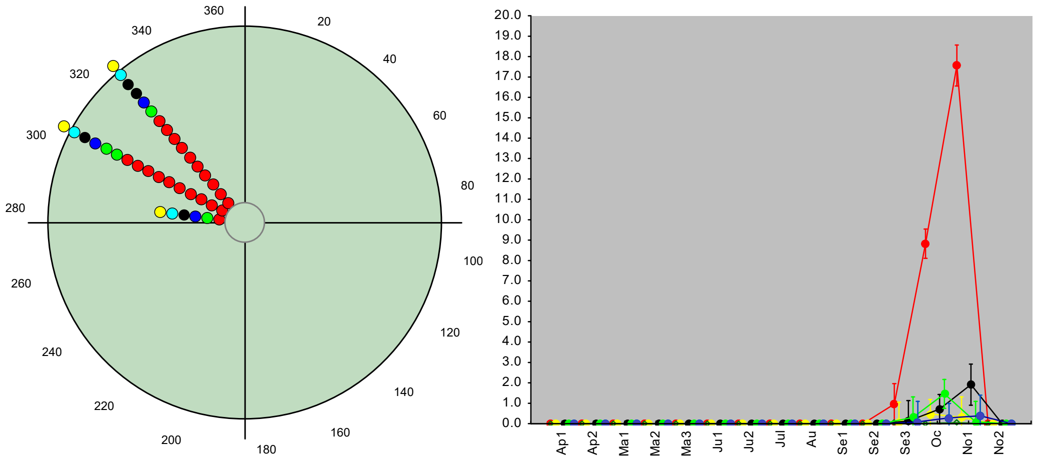

Otophorus haemorrhoidalis

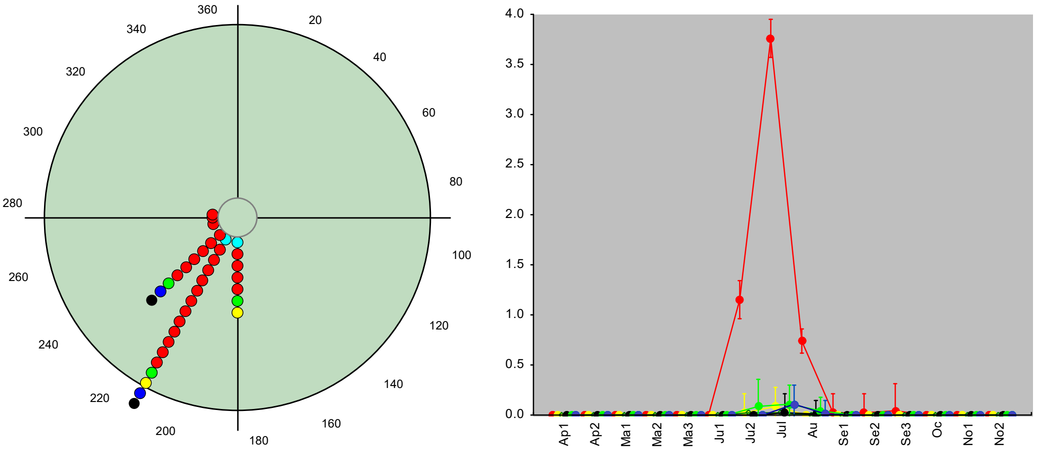

Planolinoides borealis

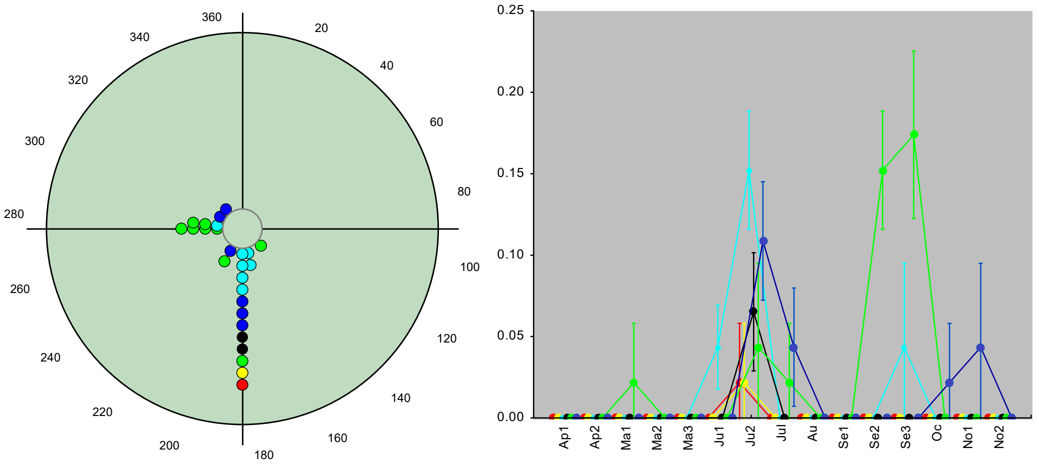

Sigorus porcus

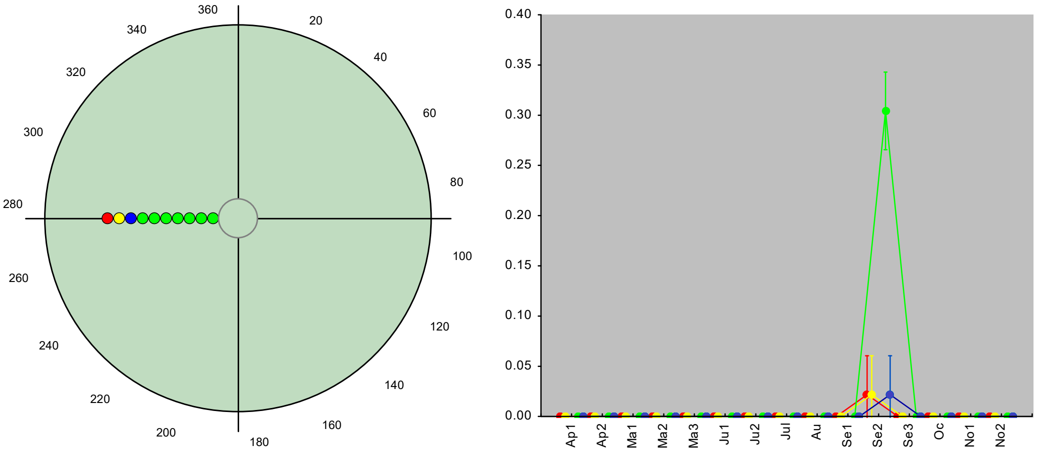

Teuchestes fossor

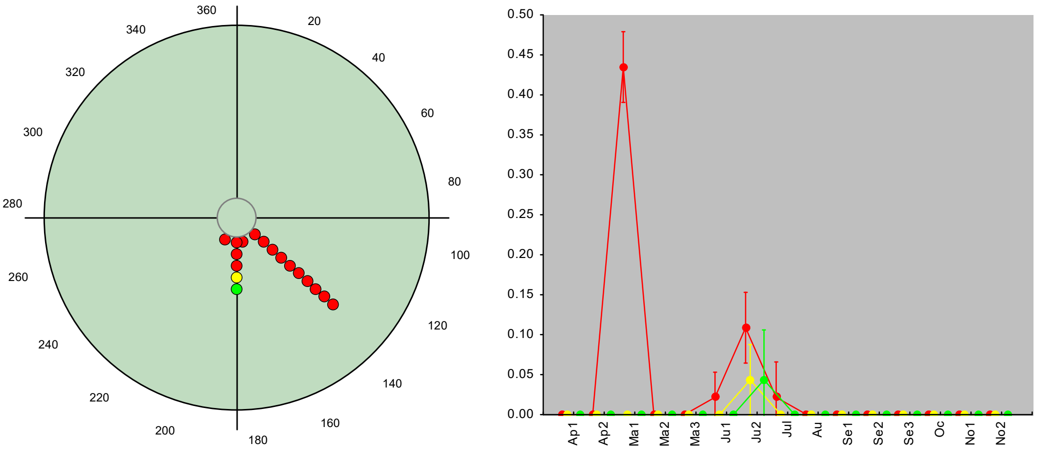

Trichonotulus scrofa

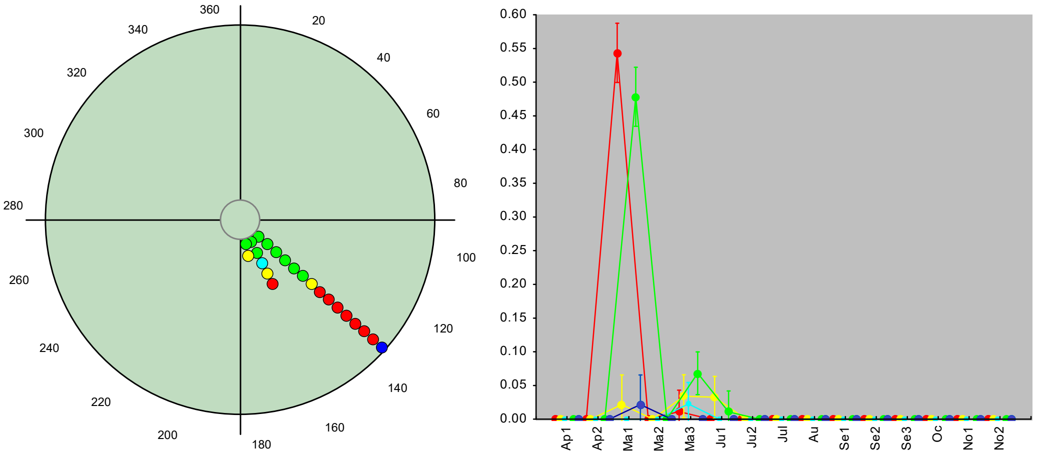

Geotrupes mutator

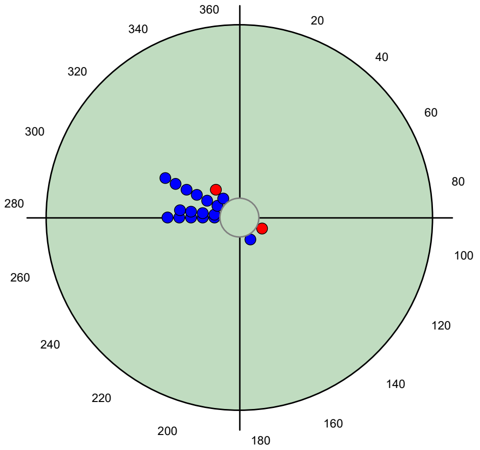

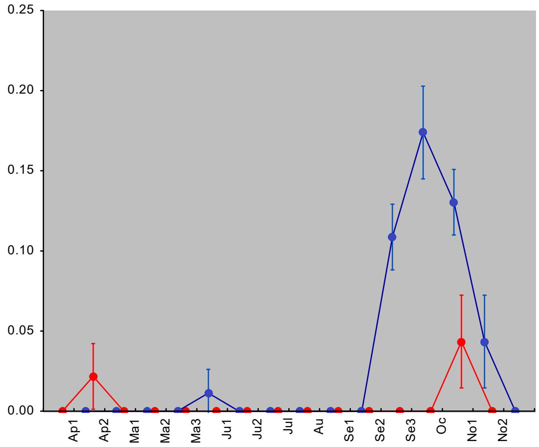

Supplement: Supplemental Information 1 — Each daily period is identified by a colour (night = black, dawn = light blue, morning = yellow, midday = red, afternoon = green, and dusk = dark blue° ) and 0° corresponds to the day of the winter solstice. Each point represents the same number of individuals. At right, Seasonal x Daily plots representing the variation in the number of collected individuals per pitfall trap (±95% confidence intervals). [file peerj-09-11786-s001.pdf]
